# Pathways and access to mental health care services by persons living with severe mental disorders and epilepsy in Uganda, Liberia and Nepal: a qualitative study

**DOI:** 10.1186/s12888-016-1008-1

**Published:** 2016-08-31

**Authors:** Rose Kisa, Florence Baingana, Rehema Kajungu, Patrick O. Mangen, Mangesh Angdembe, Wilfred Gwaikolo, Janice Cooper

**Affiliations:** 1 School of Public Health, Makerere University College of Health Sciences, P.O. Box 7072, Kampala, Uganda; 2 Transcultural Psychosocial Organisation- Uganda, P.O. Box 21646, Kampala, Uganda; 3 The Carter Center Mental Health Program in Liberia, Monrovia, Liberia; 4 Transcultural Psychosocial Organisation- Nepal, P.O.Box 8974/P.O.Box612, Baluwatar Kathmandu, Nepal

**Keywords:** Severe mental disorders, Access, Pathways to care, Post conflict countries, Qualitative study

## Abstract

**Background:**

Access to mental health care services for patients with neuropsychiatric disorders remains low especially in post-conflict, low and middle income countries. Persons with mental health conditions and epilepsy take many different paths when they access formal and informal care for their conditions. This study conducted across three countries sought to provide preliminary data to inform program development on access to care. It thus sought to assess the different pathways persons with severe mental disorders and epilepsy take when accessing care. It also sought to identify the barriers to accessing care that patients face.

**Methods:**

Six in depth interviews, 27 focus group discussions and 77 key informants’ interviews were conducted on a purposively selected sample of health care workers, policy makers, service users and care takers in Uganda, Liberia and Nepal. Data collected along predetermined themes was analysed using Atlas ti software in Uganda and QSR Nvivo 10 in Liberia and Nepal

**Results:**

Individual’s beliefs guide the paths they take when accessing care. Unlike other studies done in this area, majority of the study participants reported the hospital as their main source of care. Whereas traditional healers lie last in the hierarchy in Liberia and Nepal, they come after the hospital as a care option in Uganda. Systemic barriers such as: lack of psychotropic medicines, inadequate mental health specialists and services and negative attitudes of health care workers, family related and community related barriers were reported.

**Conclusion:**

Access to mental health care services by persons living with severe mental disorders and epilepsy remains low in these three post conflict countries. The reasons contributing to it are multi-faceted ranging from systemic, familial, community and individual. It is imperative that policies and programming address: negative attitudes and stigma from health care workers and community, regular provision of medicines and other supplies, enhancement of health care workers skills. Ultimately reducing the accessibility gap will also require use of expert clients and families to strengthen the treatment coalition.

## Background

Neuropsychiatric disorders account for about 14 % of the global burden of disease [1] and 1°2 million deaths every year [2]. However, access to care for mental disorders is inadequate for about 80 % of people [3] in low and middle income countries (LAMIC) [4]. The percentage of individuals living with severe disorders such as schizophrenia, bipolar disorder and major depressive disorder who remain untreated, is estimated to be as high as 85 % in these settings [5]. Forty one percent of English speaking adults received mental health treatment in the United States in 12 months [6], about one third of the Black Caribbean immigrants in the United States used formal mental health care services while 49.5 % of patients were diagnosed with serious mental disorder in Kenya [7]. Only 2.4 % of former combatants and 7.8 % of former non-combatants reported sufficient access to local mental health services [8]. In India, only 16 % of patients came directly to mental health professionals [9]. About 13 % of Ethiopian immigrants and refugees with a mental disorder received services [10].

On the other hand, epilepsy was estimated to account for 0 · 5 % of the global burden of disease, of which, over 85 % occurs in the 49 % of the population living in low-income and lower middle- income countries [11]. Most of these countries are in Africa and over 60 % of people with epilepsy do not access bio-medical treatment for epilepsy in LAMIC [12].

Many persons living with mental disorders delay seeking psychiatric care from formal health facilities. Their trajectories to care differ and are guided by their belief about the cause of the disorder. Community values and beliefs associate mental disorders to shame and fear thus influencing treatment seeking behavior and treatment outcomes [13]. Participants of East London First Episode Psychosis Study (ELFEPS) first sought care in descending order from: community health and social care agencies (35 %), criminal justice system (25 %), native and religious healer (21 %) [14]. In Ethiopia, half of the patients sought traditional treatment from either a religious healer 116 (30.2 %) or a herbalist 77 (20.1 %) before they came to the hospital [13].

The primary barriers to adequate mental health care are inappropriate mental health financing [15] and human resources for health [15–17]. Seventy nine percent (79 %) of African countries spend less than 1 % of their health budgets on mental health. Ghana’s per capita allocation to mental health is $0.76 [18], 0.08 % of the total budget in Nepal [19], and only 1 % of the health expenditure is spent on mental health in Uganda [20]. While developed countries have a reasonable psychiatric patient ratio, Africa has a ratio of about 0.05/100,000. There are 0.129 psychiatrists and 0.024 psychologists per 100,000 persons in Nepal [19], 0.02 psychiatrist and 146.28 nurses per 100,000 persons in Liberia [21] and only 0.08 psychiatrists: 0.04 other medical doctors: 0.78 nurses: 0.01 psychologists per 100,000 in Uganda [20]. Less than 1/3 of the health facilities provided mental health care and only 18 % of the government accredited facilities reported having mental health trained clinicians in 2010 (RBHS, 2010 [22]. Lack of awareness of the seriousness of the condition was reported by 69 % of the study participants as the main reason for delay to access professional care in India [14]. Other reasons include: belief that nothing could help and had to solve problems themselves [23], reluctance to admit to having mental health problems, perceiving seeking help as a sign of weakness or failure, denial of problems, cultural norms, recognition difficulties and lack of awareness, too embarrassed to seek help and the stigma of mental illness is a considerable barrier to mental health treatment [24]. On the other hand, cost and income disparity, unemployment, spiritual illusion thoughts about epilepsy, frustration and mental impairment, lack of availability of the same drugs on the local market were reasons cited for discontinuation of epilepsy treatment in India [25]. Patient holding traditional animistic religious and negative attitudes about bio-medical treatment, living more than 30 km from health facilities, paying for antiepileptic drugs, having learning difficulties, having had epilepsy for longer than 10 years and focal seizures were risk factors associated with epilepsy treatment gap in Kenya [26].

Due to financial and human resources scarcity, mental health has been integrated into primary health care in all the 3 countries. Such integration provides further opportunities for reducing the stigma of mental health problems [27]. Task shifting to non-specialised mental health workers has been proved to be effective in improving access thus scaling up services for persons with severe mental disorders [28]. Mental health Global Action Program (mhGAP) and mhGAP Intervention Guide (MIG) for improving accessibility of evidence based treatments for people with mental disorders [29] and evidence-based guidelines for the management of priority mental disorders by non-specialists respectively have been instituted to ameliorate the problem.

### Mental health beyond facilities project

Mental Health Beyond Facilities (mhBeF) project was implemented in three post conflict countries of Uganda, Nepal and Liberia. The project designed and is implementing a Comprehensive Community based Mental Health care package (CCMHS). The CCMHS package integrates (a) clinical component with mobile health which involves identification, referral, and clinical management of PWSMDE, (b) patient support groups to promote peer-to-peer support and provide livelihood interventions among persons with severe mental disorders and (c) anti-stigma activities intended to reduce the stigma associated with severe mental disorders and epilepsy. This study is part of a bigger formative research that the mhBeF project conducted to gain an understanding of the: views, interests, attributes and needs of the people so as to guide the design of the CCMHS package. This study specifically explored the different paths that persons with severe mental disorders and epilepsy take when seeking care and the barriers they encounter.

Although all the three countries have experienced war and one would expect the prevalent of mental disorders to be high, only a small fraction of the population that experience neuropsychiatric problems access formal mental health services. Out of the 266,608 out patients registered in Nepal in the financial year (FY = 2069/70), 529 had mental health related disorders (unpublished data, DHO Pyuthan). In Uganda, 4,034 out of 290,505 new patients registered for six months in the outpatients department in Lira district had severe mental disorders (HMIS, 2012) and between 40 and 44 % of the population have a mental disorder with depression ranging between 10 and 50 % [30–32]. In Liberia, a review of the health system estimates that as many as 11 % of adults have experienced substance use disorders, while 40 % of adults experience clinical depression, and 45 % experience Post-traumatic stress disorder [33]. This could be attributed to failure to access mental services or use of contemporary services. Whereas access to mental health services has been studied for a single serious disorder using quantitative methods and restricted to numbers, few papers have used qualitative methods to study more than one serious mental disorder. If barriers to access of services are not deeply studied for all serious mental disorders, achievement of the health related millennium development goals may be far- fetched since there is no health without mental health [1]. This paper therefore discusses barriers to access of mental health care services by persons living with severe mental disorders and epilepsy.

## Methods

Cross sectional studies involving qualitative methods of data collection and analysis were conducted between December 2012 and May 2013 in Uganda, Nepal and Liberia to: a) explore pathways to care for persons living with serious mental disorders and epilepsy (PLWSMDE), b) describe barriers that affect their access to mental health care. The study sites were: Erute south Health Sub District (HSD), Lira district in Uganda, Sinoe County in Liberia and Pyuthan district in Nepal.

Sinoe County was selected because it ranked at the bottom of a health systems assessment (2011). Government officials and partners believed that the county could benefit from improved community-based mental health services. Pyuthan and Lira districts were selected in consultation with the relevant authorities because there were no similar interventions going on during the study time.

### Erute south HSD, lira district, Uganda

Erute South HSD is located in the Eastern part of Lira with a 2012 projected population of 169,900 people [34]. There are 5 sub counties: Barr, Amach, Agali, Adekokwok and Ngetta. Erute South HSD has: one H/C IV (Amach), 5 H/C III (3 government, 2 private not for profit) and 5 H/C II (4 government and 1 private not for profit). About 31 % of the population lives within 5 km radius of health facility. The majority of the population engages in subsistence farming with 89 % living in temporary households. The most common dialect used is Langi.

### Sinoe county, Liberia

Sinoe County is located in the South Eastern region of Liberia with 17 administrative districts. The project site is located in six of the ten health districts: Greenville,Tarsue, Butaw, Dugbe, Jeadea and Kpanyan with a combined population of 49,321 people [35].

The major languages spoken are: Liberian English, Kru, Sarpo, Krahn and Bassa. It has 33 health facilities including one hospital F. J. Grante Hospital and 32 clinics. There are no health centers. Of the 32 clinics, the county health team supports 22 clinics and the rest are managed by partner health organizations support.

### Pyuthan district, Nepal

Pyuthan is located in the mid hills of Rapti zone in the mid-Western development region of Nepal. It occupies 1,365 square km. The total population of the district was 228,102 (male: 43.86 % and female: 56.14 %) in 2011. The district has one district hospital, two primary health centres, 11-health posts, and 35-sub health posts, about 40 pharmacies and three Ayurvedic health facilities.

Focus group discussions (FGDs), key informants interviews (KIIs) and in depth interviews (IDIs) guides with open-ended questions were used to collect data from purposively selected samples who were 18 years old and above. The core interview guides were developed by “mhBeF” consortium according to themes that were determined by the CCMHS package component leads before data collection. The core interview guides were then developed based on those themes by “mhBeF” consortium. Necessary adaptations were made for each country after pre- testing. They were also punctuated with probes to further obtain accurate information. Open-ended questions were used to explore the experiences of PLWSMDE and their caregivers and also to elicit a wide range of perceptions and needs from persons living with severe mental disorders and epilepsy, their caregiver and other major stakeholders as shown in the Table 1.

**Table 1:** Showing study participants and qualitative methods used to collect data in the 3

	FGD	KII	IDI
Country			
Uganda	Village Health teams, community and religious leaders, traditional healers, teachers (primary and secondary) and care givers. Each group had at least 8 members	Specialists and policy makers (Chief Administrative Officer (CAO), Assistant CAO, CDO and DHO, LRRH director, in charge mental health unit, district mental health focal person, district pharmacist, secretary for health, Non- governmental Organizations (NGO) administrators, health care workers (HCWs)	PWSMDE (2 patients with, schizophrenia, 2 epilepsy and 2 Bipolar disorders)
Liberia	Community, Banna Town, family members of service users, health facility	Dispenser/Nurse, police officer, Nurse/District Health Officer, Service Head/ Psychosocial Counselor, Pharmacist, Logistics Officer, Nurse Supervisors, Nurses, Health administrator, mental health clinician, NGOs, service users, religious, community, traditional and policy maker/ disability union leaders, family member of service user, community health volunteers	None
Nepal	Community leaders, Teachers, mother groups, Auxiliary nurse midwives (ANM) service users, government health facility in charges and Female Community Health Volunteers	Policy makers, Primary Health Care workers, Female Community Health Volunteers (FCHVs), Pharmacists, Political leaders, traditional healers, herbalists, NGO workers, Teachers, VDC Secretary, service users and service users‟ care givers.	None

Prior to data collection, site visits were made in all the three countries. Meetings were held with some district officials, community members and patient representatives to introduce the project, raise awareness about the study and get their buy-in and ownership.

Participants were approached differently: those with mobile phones were called to fix an appointment prior to face-to-face interview. Those without mobile phones were approached physically. Study participants from health organizations were recruited through a home visit. Work place visits were used for health care workers in Nepal. In Uganda, persons with severe mental disorders and epilepsy were recruited using clinic registers with the assistance of Psychiatric Clinical Officers. Caregivers were recruited from the waiting room of the mental health unit of Lira Regional Referral Hospital, and the health care workers and village health team members were recruited from health facilities. The rest of the study participants were recruited from the nearby communities and converged at the Sub County headquarters. Whereas written consent was obtained from KII and IDI literate participants, thumb prints and verbal consent were used for illiterate participants and those in the FGDs respectively.

Six IDIs, 12 FGDs each composed of 7–10 participants and 29 KIIs were held in a quiet private environment in Uganda. Twenty-two KIIs and six FDGs each consisting of 5–8 participants were conducted in Liberia and 26 KIIs and nine FGDs of at least six participants each were conducted in Nepal. The number of interviews and FGDs were conducted until additional ones could not generate new information. Homogeneity was observed in terms of occupation and location in FGDs. All participants were selected because we anticipated to obtain useful information that would guide the implementation of the mhBeF interventions. All data was audio recorded along with note taking by extensively trained gender balanced degree holder research assistants. The research assistants were trained on establishment of rapport with participants prior to administering the interview. They were closely supervised and daily reviews to discuss field experiences were conducted.

### Data management and analysis

Data collected from English speaking participants were transcribed while that from nonEnglish speaking participants were transcribed and translated to English. This applied research findings aims to influnece the plan and policies of respective countries through recommendations often within a short time frame. There is an increasing trend of using framework analysis methodology in contrast to grounded theory which is developed to be is used in the context of applied policy research. Framework analysis (FA) allows for data collection, management and interpretation in a sequential fashion. Framework Analysis applies a three-pronged approach to the data, examining the data by themes, by type of respondent and by explanatory models available. The research team sought to understand what questions needed to be answered to inform how a project to deliver comprehensive mental health services should be organized. What barriers would need addressing and who would be important drivers of successful implementation? Data was collected from important actors in communities, themes that emanated from discussions and factors explained the context for service delivery. So, the data was analyzed thematically using the framework analysis method [36] It was cleaned, merged with field notes to make final transcripts and coded. A preliminary coding framework was developed on the basis of prior themes and emerging themes. Codefilter was developed putting the highlighted data in the categories that make sense from the interviews data. A prelimanry coding framework was pilot tested on 25 % randomly selected data analyzed by two different experienced researchers in each country who then adopted and made changes where necessary and the final coding framework was determined. This final coding framework was applied to all data sets. For coding and charting software Atlas. Ti was used in Uganda while Liberia and Nepal used QSR NVivo 10 software.

## Results

This paper presents results from Uganda, Liberia and Nepal on the various paths that PLWSMDE take to seek mental health care and the barriers they face in accessing formal mental health care. The majority of the study participants in Liberia were male with college education level and between 22 and 63 years. Langi females who are married dominated the Ugandan study participants. Key informant interviews and IDIs on average lasted for 30 to 45 min while an FGD went for one hour on average.

### Pathways to care

All participants reported being guided by beliefs when seeking mental health services. Many of them went to hospital when the first choices do not yield positive results. This practice often results into late reporting hence the worsening of illness. The three most common reported sources of mental health care for PLWSMDE in Uganda in descending order are: health facility especially the Lira mental health unit, traditional healers and witch doctors and places of worship. Aside from the hospital, PLWSMDE in Liberia, were more likely to seek care from religious leaders compared to traditional healers. Participants reported that the choice of care depended upon what the family’s perceptions of the cause of illness, if they felt witchcraft was the cause of mental illness, they were more likely to seek care from a traditional healer. In Nepal, study participants reported that community people would rather receive treatment from traditional healers than opt for medical treatment. Generally, community people do not seek treatment for general mental illness unless the problem is severe. When they do seek care it is outside the community as there are no available services for severe mental disorders. According to study participants, economically strong families seek help from different parts of the country and outside of the country such as in India.

The following illustrates participants’ pathways to care in their own words:

“It depends on the cause, what the family member thinks is the cause. If some family member starts to have mental illness, and I think that the reason for this is because of witchcraft I will want to take that person to country doctor. Or (if there is) some spiritual cause I will say let me carry the person to the church so they can pray for the person, so what is bothering the person, that demon or so can leave the person. So it depends on what I think is the cause of the problem.” (FGD 04 – Health Facility, Liberia)

“Visiting a health facility for mental health care is not very common. Our people believe that mental illness is from witchcraft: it is demonic. So they are now more in the church than the health facilities … others go to the witchdoctors to consult and take some local medicines. The highest percentage (of people with mental illness) believes somebody is out there using the demons to torment them” (KII-Policy maker, Uganda)

“People believe more on traditional healers because people say “bojulageko” to the person suffering from mental problems. So they first go to traditional healers and then only come for medication and if they do not recover here, they go outside of the district e.g Butwal, Nepalgunj”(FGD- Health Facility In charges, Nepal)

Participants cited a number of barriers to access mental health care services in the three countries. These are presented in the Table 2.

**Table 2:** Showing barriers to access mental health care services cited by study participants from the 3 countries

Barrier	Nepal	Liberia	Uganda
Lack of awareness	✓	✓	✓
Economic burden	✓	✓	✓
Familial			
Delay in family decision for seeking care	✓		
Myths and misconception regarding mental health problems	✓		✓
Social stigma and discrimination	✓	✓	✓
Lack of social support			✓
Poverty	✓		
Unwillingness of patients to take prescribed medicine (e.g. due to fear of side effects, severity of illness, and lack of support at home)			✓
Low regard of the mentally ill: negligence of caregivers/family members/community members (considered useless)			✓
Recurrence of the illness			✓
Systemic			✓
Inaccessible mental health services due to geographical constraints	✓	✓	✓
Negative attitudes of health workers	✓	✓	✓
Lack of patient follow-up			✓
Lack of mental health medicines,	✓	✓	✓
Change of service providers			✓
Inadequate mental health specialists	✓	✓	✓
Overcrowding at the health facilities			✓
Long distances to the health centers		✓	✓
Fear of PLWSMDE by other patients and caregivers at the health centre			✓
Community			✓
Traditional beliefs about mental illness (witchcraft, curses, incurable), leading to preference for traditional remedies	✓		✓
Lack of awareness	✓	✓	✓
Economic burden	✓	✓	✓
Lower cost of services and more flexible terms offered by traditional healers			✓
Unwillingness of some patients to go the health centers, sometimes due to lack of insight			✓

Among the barriers identified that were unique to Uganda’s Lira district included: overcrowding at health facilities, more competitive rates and terms of services by traditional healers, fear of persons with mental health conditions by other patients at health facilities: and greater preference for traditional healing remedies. Lack of social support and lack of patient follow-up were also reported in Uganda. Liberia reported no barriers unique to its setting. All three countries identified lack of awareness, economic burden, social stigma and discrimination, geographical inaccessibility and long distance to facilities. Nepal and Liberia both reported lack of medicines and inadequate number of mental health specialists as barriers.

Lack of awareness about mental health services was commonly mentioned by all the participants as one of the barriers to seeking mental health services.

“Most people in our community do not know where to seek help, like for me it is some people who told me to come to town next to the stadium where I could get help. In the community when you get an attack people actually fear you thinking that the disease can also transfer to them; so when it attacks you they give you traditional medicine to take.” (FGD of caregivers- Uganda)

“Our community does not believe that mental problem is also a health related problem rather they believe it due to lack of awareness, many family take mental problem as a burden and they behaves negatively towards their ill member. Therefore, the important thing they need is awareness about mental problem and its treatment service.” (KII-Teacher Nepal)

An inadequate mental health clinicians and medicines were universally cited by participants in all the study sites. It is reaffirmed by the quotes below:

“We only get Diazepam, which is given for our facility; we don't have actual medicine for mental health. We only do counselling, like when we find (out) that the patient is combative, we serve Diazepam” (KII 14 – HF/Nurse- Liberia)

“We have (a) low staffing problem. We don’t have sufficient nurses or midwives to sit down (you know) because dealing with mental illness or mental health problem, it has to take long time, and you can’t really do it in a hurry.” (KII 09 – HF/Midwife- Liberia)

“There are no mental health services available at PHCC but there are some hospitals with mental health specialist. There is post of psychiatric doctor at the zonal and regional hospital and the services are available but district hospital and PHCC do not provide mental health service” (KII-MoHP, Section Officer Nepal)

“There are no drugs at the health centres; we have the problem of walking long distances to bring our patients here… “(FGD of caregivers-Uganda)

“Sometimes you go in the morning and come back in the evening with no proper treatment because people are many and sometimes they say ‘today we don’t have enough drugs’. You are told to come back the next day… But you know the sickness cannot wait for another day; it keeps on progressing.” (FGD of community leaders- Uganda)

### Economic burden

Although participants from Uganda and Nepal cited economic burden as a barrier to seeking mental health care from formal health facilities, those in Liberia reported that when compared to formal services traditional and religious healers extracted higher prices. So instead of high costs driving them away from the formal services, it was the opposite.

“For [us] the hospital, it is free. You only just have to come, it is the matter of coming, it leaves (its left) with your effort for coming. But these traditional people, they charge you Liberian dollars (LD) $1000, some LD $3000, sometimes LD $4000.” (FGD – 05 – Community- Liberia)

People suffering from mental illness thought they would be mentally ill for the rest of their lives and since treatment of mental illness requires a long period of time, it is also economically unviable for many to treat the illness. Lack of money to pay for the necessities such as medicines not available in the health facilities, transportation and bicycle parking fees presented barriers for the caregivers and the patients.

“Financial constraint is another issue for us. We have to walk long distances and remember walking with these patients is not easy as they are unpredictable in behaviour.” (FGD of caregivers-Uganda)

“Due to low economic status people are unable to go out of district for treatment as well as they are unable to afford medicine for long time. Some people are careless and they do not go for treatment.” (KII-Teacher-Nepal)

“Like (if) someone has a mental health problem in Gbana town, the last district in Sinoe, to refer them to Greenville here, you know it can be expensive, this is a problem.” (KII- Community - Liberia)

### Social isolation

Many families do not offer social support to their family members. They often delay seeking care and many times patients are left to go to the health centres unaccompanied, even when they are unable to cope on their own due to the severity of illness.

“Sick people are sick. They are not capable to understand these things. But their family does not take them to a health facility for treatment because they are afraid that others may know their problem and react against them. Families hide their problems because community people have negative perceptions of people with mental problems. Families who are rich take them to a health facility without other people knowing about their problem. Only poor people remain confused so everybody knows their problem” (KII-Pharmacist- Nepal)

“You know some of them don’t talk well. So they find difficulties in expressing themselves. Some of them cannot even talk. When some of them talk, saliva drops from their mouths. Others like me talk endlessly… So it is very difficult (for the health worker) to know our problems” (IDI with a PWSMDE- Uganda)

All study participants mentioned stigma from the community as a major obstacle to accessing care,. Stigma was discussed in the form of: avoidance of contact and association with patient, caregivers and patient’s family: not showing love or care to patients (neglecting them): unkind comments about patient, caregiver and family: and calling patients derogatory names. Participants noted that stigma stops clients from going for treatment and joining patients’ groups for fear of being easily identified.

“Mental disorders carry social stigma along with it in the community. So, people don’t want to disclose their problem in the community. Due to this people show consciousness and they are afraid community people may know their problem and with this conception they stop revisit the service center” (KII- MCHW, Nepal)

“Some in the community fear people with epilepsy… They believe that it is transmitted by staying with or being near that person… people end up running away from the patient… fitting, falling down, convulsing and there is nobody to attend to him… even the home itself will be stigmatized… people will say … that home has a disease: they call it a bad disease” (Health worker in a HC III-Uganda)

## Discussion

This study provides evidence on barriers to mental health care experienced by persons living with severe mental health disorders and epilepsy in the three post-conflict countries. Results presented come from formative studies undertaken to inform the design of the interventions of the Comprehensive Community-based Mental Health Care package for the mhBeF project. Responses from participants show that many pathways are followed when accessing mental health services in the three countries. Results from our analysis can be categorized into three major types of barriers to access: systemic or institutional, familial and community. Among the systemic barriers to mental health services cited by participants include: lack of medicines, inadequate mental health specialists and services, change of health care providers, negative attitudes of health care workers, their fear for PWSMDE, absence of follow-up services, overcrowding and geographical inaccessibility seriously impede access to mental health services. The absence and shortage of medicine can be explained by a “push system” where the government supplies a minimum medicines package to lower level facilities, but does not provide health care workers with opportunities to request for medicines based on need. In addition, in all countries the lack of adequate medicines to treat mental illness and epilepsy remain a barrier to care and often the selection of the health facility option. The problems with provider shortages and provider skills’ match can be explained by a ban on recruitment of health care workers in Uganda, as well as, staff turn-over. In Liberia and Nepal the lack of trained mental health workers remains a significant problem. The lack of psychotropic medicines in all three countries as a finding is consistent with a study in India where lack of medicines led to discontinuation of epilepsy treatment [25]. Geographical inaccessibility is attributed to absence of mental health specialists and medicines in the nearby lower level facilities, thus patients have to trek longer distances to the Regional Referral Hospital (RRH). This also explains the overcrowding at the RRH. Negative attitudes and fear of PWSMDE by health care workers could be due to lack of adequate training in severe mental disorders. Geographical inaccessibility concurs with findings from a study conducted among young people with depression [37], mentioned as one of the risk factors associated with epilepsy treatment gap [26] and in disagreement with a retrospective cross-sectional study in California where the availability of epilepsy centers did not influence access to specialized epilepsy care in people who had private insurance [38].

The second main set of barriers is related to family. Some families are unaware and uncertain about the availability and location of mental health services. Others ignore their family members who are sick resulting in delay to seek help as well as failure to comply to treatment. Similar findings were reported in India where families were ignorant of the availability of services, cost of services and lack of transport [39].

Communities also can discriminate and direct stigmatising statements towards PWSMDE and their families and this deters them from seeking mental health services. Persons with severe mental disorders and epilepsy are regarded as useless, neglected by caregivers, family members and even their community. This is exacerbated by the myths and misconceptions surrounding the cause and treatment outcome of mental illnesses. Many people regard mental illnesses to be caused by witchcraft and incurable to western medicines. This drives them away from seeking professional help [40, 41] and leads to poor adherence to prescribed medicines for those who attempt to go there [13, 42]. However, stronger discriminative intentions were reported not to necessarily prevent professional service use in Finland in case of a serious condition and having realistic views about medication [13, 43]. The difference could be attributed to use of non-standard tools that don’t warrant comparison in Finland and quantitative methodology in both.

The paper makes several contributions to the literature and the mental health policy field. It provides information on three different post-conflict low-income countries with similar experiences in mental health access for service users. It uses different qualitative methods of data collection and incorporates common mental health disorders. The qualitative methodology used in this study provides a deeper understanding of barriers to accessing mental health care services. While the qualitative methodology provides depth to our findings, these are not generalisable. The results are subjected to selection bias since some participants (PWSMDE and their caregivers) were selected from the mental health unit, and the community mobilizers could have picked individuals they thought would give impressive answers. Finally, it draws on a unique south-to-south collaboration that bring insights that point to commonalities among low- and middle income countries irrespective of geography.

## Conclusion

Access to mental health care services by persons living with severe mental disorders and epilepsy is remains low in these three post conflict countries. The reasons contributing to it are multi-factored ranging from systemic, familial, community and individual. It is imperative that policies and programming address: negative attitudes and stigma from health care workers and community, regular provision of medicines and other supplies, enhancement of health care workers skills. Ultimately reducing the accessibility gap will also require use of expert clients and families to strengthen the treatment coalition.

## Abbreviations

CCMHS: Comprehensive community based mental health services package
DHO: District Health Officer
mhBeF: Mental health beyond facilities project
PWSMDE: Persons living with severe mental disorders and epilepsy
RRH: Regional referral hospital
VDC: Village development committee
